# Intraoperative PEEP Strategy and Postoperative Pulmonary Complications in Obese Patients: A Randomized Trial with Exploratory Analysis of Smoking Status

**DOI:** 10.3390/medsci14020284

**Published:** 2026-05-31

**Authors:** Luca Gregorio Giaccari, Simona Brunetti, Francesco Coppolino, Maria Caterina Pace, Maria Beatrice Passavanti, Vincenzo Pota, Pasquale Sansone

**Affiliations:** 1 Department of Woman, Child and General and Specialized Surgery, University of Campania “Luigi Vanvitelli”, Piazza Luigi Miraglia 2, 80138 Naples, Italy; lucagregorio.giaccari@gmail.com (L.G.G.); simona.brunetti@studenti.unicampania.it (S.B.); francesco.coppolino@unicampania.it (F.C.); mariacaterina.pace@unicampania.it (M.C.P.); mariabeatrice.passavanti@unicampania.it (M.B.P.); vincenzo.pota@unicampania.it (V.P.); 2 Service of Anesthesia and Intensive Care, Vito Fazzi Hospital, 73100 Lecce, Italy

**Keywords:** obese, smoking, high PEEP, recruitment maneuvers, postoperative pulmonary complications, intensive care unit

## Abstract

**Background**: Obesity increases the risk of postoperative pulmonary complications (PPCs), and active smoking may further amplify this risk. Whether smoking status identifies a subgroup of obese surgical patients with differential PPC risk or a different response to intraoperative Positive End-Expiratory Pressure (PEEP) strategy remains unclear. We evaluated whether smoking status influences PPCs and modifies the effect of intraoperative PEEP strategy in obese patients undergoing surgery. **Methods**: In this single-center randomized trial, 95 obese surgical patients were assigned to either a low-PEEP strategy (4 cmH_2_O without recruitment maneuvers) or a high-PEEP strategy (12 cmH_2_O with recruitment maneuvers). The primary endpoint was PPC incidence within 5 postoperative days in the overall randomized population. Smoking status was recorded at baseline, and pre-specified exploratory subgroup analyses assessed PPC incidence according to smoking status and the smoking-by-PEEP interaction. **Results**: The overall incidence of postoperative pulmonary complications (PPCs) was 8.9% in the low-PEEP group and 8.0% in the high-PEEP group (*p* > 0.05). Among smokers, complications occurred in 18.2% in the low-PEEP group and 11.8% in the high-PEEP group. For non-smokers, rates were 5.9% and 6.1%, respectively. No statistically significant differences were observed. **Conclusions**: Active smoking was associated with a numerically higher incidence of PPCs in obese patients; however, this finding was not statistically significant. The high-PEEP strategy with recruitment maneuvers did not reduce PPC incidence compared with the low-PEEP strategy. **Trial Registration**: Approval number 003208/2016.

## 1. Introduction

Obesity and smoking are associated with increased morbidity and mortality. According to the World Health Organization (WHO), more than 1.9 billion adults were overweight in 2016, of whom over 650 million were classified as obese [1]. Globally, more than 1.1 billion people smoke tobacco, and at least 367 million use smokeless tobacco products [2]. The prevalence of smoking increases with higher body mass index (BMI) [3].

Both obesity and smoking are known to cause significant adverse health effects, including cancer, cardiovascular disease, and respiratory disorders [2,4]. Obesity is linked to a higher risk of death; in 2015 alone, it was responsible for 4.42 million deaths [4]. During the same period, smoking accounted for approximately 11.5% of all deaths worldwide [2].

These conditions significantly raise the risk of preoperative, intraoperative, and postoperative surgical complications [5,6]. Postoperative pulmonary complications (PPCs) refer to respiratory complications that occur after anesthesia and surgery. These may include clinical conditions such as pneumothorax, atelectasis, pleural effusion, bronchospasm, pneumonia, weaning failure, prolonged mechanical ventilation, respiratory failure, and the need for reintubation. PPCs are common, difficult to predict, and have significant consequences for patient outcomes [7]. Their incidence in the general surgical population ranges from 2% to 5.6% [8].

In obese and patients who smoke, the combination of reduced functional residual capacity, increased chest wall impedance, and smoke-induced airway inflammation predisposes patients to early alveolar collapse during general anesthesia. The application of a high-Positive End-Expiratory Pressure (PEEP) strategy, along with alveolar recruitment maneuvers, may theoretically counteract these effects by stabilizing airway patency, improving gas exchange, and preventing atelectasis in already compromised lungs.

Obesity increases the risk of atelectasis and impaired respiratory mechanics during general anesthesia [9]. Perioperative atelectasis is a major cause of both intra- and postoperative hypoxemia and pulmonary infections [9]. Tobacco use contributes to sputum retention, pneumonia, and respiratory failure [10].

Evidence supporting specific intraoperative ventilation strategies to reduce PPCs in these comorbidities is currently limited. The PROBESE study found that, among obese patients undergoing surgery under general anesthesia, a mechanical ventilation strategy with high-positive end-expiratory pressure (PEEP) strategy and alveolar recruitment maneuvers did not reduce the incidence of PPCs compared to a strategy with lower levels of PEEP [11]. Similarly, the PROVHILO study, which included patients at risk for PPCs after abdominal surgery, randomized patients to either low PEEP without recruitment maneuvers or high PEEP with recruitment maneuvers. No significant differences were observed between the two groups in terms of PPC incidence during the first five postoperative days [12].

**Figure 1:**
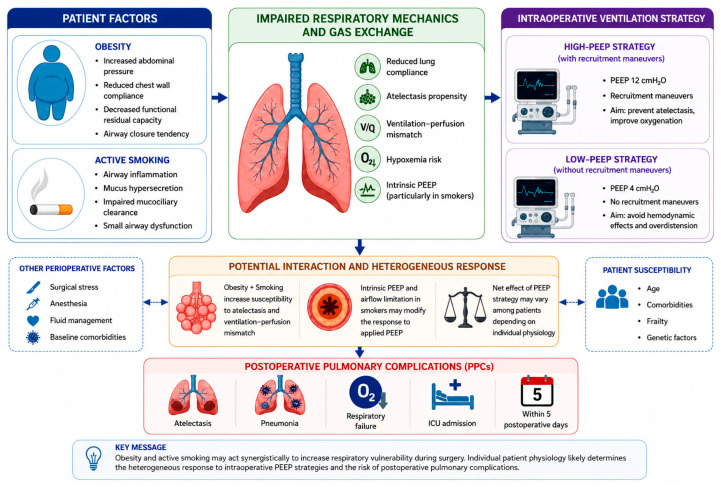
Obesity, Smoking, and PPC Risk.

Although obesity is an established risk factor for PPCs and smoking is associated with adverse postoperative respiratory outcomes, it remains unclear whether active smoking identifies a particularly high-risk subgroup among obese surgical patients or modifies the effect of intraoperative PEEP strategy. To date, no randomized study has specifically explored whether smoking status modifies the effect of intraoperative PEEP strategies in obese patients. The proposed pathophysiological relationship among obesity, active smoking, impaired respiratory mechanics, and PPCs is summarized in Figure 1.

Aims. The primary endpoint was the incidence of PPCs within the first five postoperative days, comparing high- versus low-PEEP ventilation strategies in the overall randomized population. Exploratory endpoints included assessment of PPC incidence according to smoking status and evaluation of the interaction between smoking status and PEEP strategy.

## 2. Materials and Methods

**Design**. This study was conducted at “Luigi Vanvitelli” University Hospital (Naples, Italy) between July 2016 and February 2018. The study followed the CONSORT guidelines [13], and the reporting checklist is provided in the Supplementary Materials. The study protocol was approved by the local ethics committee (approval number: 003208/2016) and conducted in accordance with the Declaration of Helsinki. All participants provided written informed consent prior to inclusion.

**Endpoints.** The primary endpoint was the incidence of postoperative pulmonary complications (PPCs) within the first five postoperative days, comparing high-PEEP versus low-PEEP strategies in the overall randomized population. Exploratory endpoints included assessment of PPC incidence according to smoking status and evaluation of the interaction between smoking status and PEEP strategy. Secondary endpoints included the rate of postoperative ICU admission.

**Inclusion and Exclusion Criteria.** Inclusion criteria were: 1. patients scheduled for surgery under general anesthesia; 2. medium-to-high risk for PPCs, defined as an ARISCAT score ≥ 26; 3. body mass index (BMI) ≥ 35 kg/m^2^; 4. expected surgical duration ≥ 2 h. Only elective surgical procedures were included in the study, allowing complete preoperative assessment and ARISCAT score calculation before enrollment.

Smoking status was routinely assessed during the pre-anesthetic evaluation through direct patient interview and review of medical history. Patients were classified as active smokers or non-smokers; information on pack-years, former smoking, and timing of preoperative smoking cessation was not systematically collected.

The ARISCAT (Assess Respiratory Risk in Surgical Patients in Catalonia) score has demonstrated reliability in identifying patients at increased risk for PPCs [14].

Exclusion criteria were: 1. age < 18 years; 2. previous lung surgery; 3. persistent hemodynamic instability or intractable shock; 4. history of chronic obstructive pulmonary disease (COPD); 5. chemotherapy or radiotherapy within the two months prior to surgery; 6. severe cardiac disease (NYHA class III or IV), acute coronary syndrome, or persistent ventricular tachyarrhythmia; 7. invasive mechanical ventilation > 30 min within the last 30 days; 8. Pregnancy (excluded via medical history and/or laboratory testing); 9. history or suspicion of acute respiratory distress syndrome requiring prolonged postoperative ventilation; 10. severe pulmonary arterial hypertension (systolic pulmonary artery pressure > 40 mmHg); 11. head trauma or brain tumor; 12. neuromuscular disorders; 13. requirement for intraoperative prone or lateral decubitus positioning; 14. requirement for single-lung ventilation; 15. cardiac surgery; 16. neurosurgery; 17. scheduled postoperative reintubation; 18. enrollment in another study or refusal to provide informed consent.

**Procedure.** Eligible patients received volume-controlled mechanical ventilation with a tidal volume of 7 mL/kg predicted body weight and were randomized into two groups:

1. Low-PEEP strategy group: PEEP of 4 cmH_2_O without alveolar recruitment maneuvers.
2. High-PEEP strategy group: PEEP of 12 cmH_2_O with alveolar recruitment maneuvers.

For recruitment maneuvers, tidal volume and, if necessary, PEEP were gradually increased until a plateau airway pressure of 40–50 cmH_2_O was reached. All patients received the lowest possible fraction of inspired oxygen (FiO_2_ ≥ 0.4) to maintain peripheral oxygen saturation (SpO_2_) above 92%. Each group was further stratified into smokers and non-smokers.

Randomization was conducted via a dedicated website and was stratified based on ARISCAT scores (≥26) to ensure balanced risk distribution between the medium- and high-risk groups.

PPCs were evaluated for five days postoperatively and included the following complications: aspiration pneumonia, bronchospasm, mild/moderate/severe respiratory failure, ARDS, lung infection, atelectasis, pulmonary edema, pleural effusion, and pneumothorax.

Outside the context of the present trial, intraoperative ventilatory management in obese surgical patients at our institution was not fully standardized and was left to the discretion of the attending anesthesiologist. In general, low-to-moderate PEEP levels (approximately 4–6 cmH_2_O) were commonly applied, while recruitment maneuvers were selectively performed according to intraoperative oxygenation and respiratory mechanics.

**Statistical Analysis.** All analyses were conducted according to the intention-to-treat principle. Continuous variables were summarized as mean ± standard deviation (SD) or median (interquartile range), as appropriate based on data distribution. Categorical variables were reported as counts and percentages.

Between-group comparisons for continuous variables were performed using Student's *t*-test or Mann–Whitney U test, as appropriate. Categorical variables were compared using the chi-square test or Fisher’s exact test, depending on expected cell counts.

In addition to hypothesis testing, effect estimates with corresponding 95% confidence intervals (CIs) were calculated for all main outcomes. For the primary endpoint (postoperative pulmonary complications, PPCs), absolute risk differences and relative measures of effect (risk ratios or odds ratios) were reported.

Given the limited number of events, exact methods were used when appropriate to improve estimate reliability. Exploratory subgroup analyses were performed according to smoking status (active smokers vs. non-smokers). The study was not powered or specifically designed to detect interactions between smoking status and PEEP strategy, and this interaction was formally assessed using an interaction term in a regression model.

To explore the robustness of the findings, sensitivity analyses were performed using multivariable logistic regression, adjusting for clinically relevant baseline variables, including age, sex, body mass index (BMI), ARISCAT score, and type or duration of surgery, when available.

No formal sample size calculation was performed for subgroup analyses based on smoking status; therefore, these analyses were considered exploratory and hypothesis-generating. A two-sided *p*-value < 0.05 was considered statistically significant, without adjustment for multiple comparisons.

All statistical analyses were performed using SAS version 9.4 (SAS Institute Inc., Cary, NC, USA).

## 3. Results

A total of 95 patients were enrolled and randomized, with 45 assigned to the low-PEEP strategy group and 50 to the high-PEEP strategy group. All randomized patients were included in the intention-to-treat analysis.

Baseline demographic and clinical characteristics were comparable between groups (Table 1). Sex distribution was similar, with 14 males and 31 females in the low-PEEP strategy group and 20 males and 30 females in the high-PEEP strategy group. The mean age was 41.18 ± 10.26 years in the low-PEEP strategy group and 39.54 ± 11.77 years in the high-PEEP strategy group (*p* = 0.24). Body mass index (BMI) was also comparable (45.74 ± 6.17 vs. 45.23 ± 5.66 kg/m^2^; *p* = 0.34).

Obesity class distribution was similar between groups, with most patients classified as class III obesity (25/45 in the low-PEEP strategy group and 34/50 in the high-PEEP strategy group). The mean ARISCAT score was 35.87 ± 4.78 in the low-PEEP strategy group and 38.50 ± 9.50 in the high-PEEP strategy group (*p* = 0.05). Although the ARISCAT score was numerically higher in the high-PEEP strategy group, exploratory adjusted analyses yielded results consistent with the primary analyses.

Active smoking was reported in 11 patients (24.4%) in the low-PEEP strategy group and 17 patients (34.0%) in the high-PEEP strategy group, corresponding to 28 of 95 patients overall (29.5%).

Although baseline characteristics were generally balanced between groups, the ARISCAT score and smoking prevalence were numerically higher in the high-PEEP group.

Ventilatory management differed according to protocol allocation. Patients in the high-PEEP strategy group received a PEEP of 12 cmH_2_O with recruitment maneuvers, whereas those in the low-PEEP strategy group received a PEEP of 4 cmH_2_O without recruitment maneuvers. No clinically relevant differences were observed in other intraoperative variables between groups.

Postoperative pulmonary complications (PPCs) within 5 days occurred in 4 of 45 patients (8.9%) in the low-PEEP strategy group and in 4 of 50 patients (8.0%) in the high-PEEP strategy group. There was no evidence of a difference in PPC incidence between the two ventilation strategies. The relative risk (RR) of PPCs in the high-PEEP strategy group compared with the low-PEEP strategy group was 1.11 (95% CI 0.30–4.18), with an absolute risk difference of 0.9 percentage points. The wide confidence interval indicates that clinically relevant differences between groups cannot be excluded. 

In exploratory analyses, smokers showed a numerically higher incidence of PPCs compared with non-smokers.

Among active smokers, PPCs occurred in 2 of 11 patients (18.2%) in the low-PEEP strategy group and in 2 of 17 patients (11.8%) in the high-PEEP strategy group. Among non-smokers, PPCs occurred in 2 of 34 patients (5.9%) in the low-PEEP strategy group and in 2 of 33 patients (6.1%) in the high-PEEP strategy group.

When pooled across ventilation strategies, PPCs occurred in 4 of 28 smokers (14.3%) and in 4 of 67 non-smokers (6.0%). Smokers had a higher, although not statistically significant, risk of PPCs compared with non-smokers (RR 2.38, 95% CI 0.64–8.85).

No statistically significant interaction between smoking status and PEEP strategy was observed.

Baseline characteristics of smoking patients were similar between groups (Table 2). Age, BMI, obesity class distribution, and ARISCAT score did not differ meaningfully, and no clinically relevant imbalances were identified.

No clinically meaningful differences in baseline characteristics of smoking patients were observed between ventilation strategy groups.

The distribution of specific PPCs was similar between groups (Table 3). Observed complications included respiratory failure (*n* = 6), pleural effusion (*n* = 2), atelectasis (*n* = 2), and acute respiratory distress syndrome (ARDS; *n* = 2).

Given the small number of events, no formal comparisons for individual PPC components were performed.

No postoperative extrapulmonary complications related to ventilation strategy were observed. Specifically, there were no cases of hepatic failure, renal failure, acute myocardial infarction, disseminated intravascular coagulation, extrapulmonary infection, systemic inflammatory response syndrome, sepsis, or coma.

No patient required admission to the intensive care unit within 30 days after surgery.

In exploratory multivariable analyses adjusting for age, sex, BMI, ARISCAT score, and surgical characteristics (when available), results were consistent with the unadjusted analyses, showing no evidence of a difference between ventilation strategies and no robust association between smoking status and PPCs.

## 4. Discussion

In this single-center randomized trial including obese surgical patients, a high-PEEP ventilation strategy with recruitment maneuvers was not associated with a reduction in postoperative pulmonary complications (PPCs) compared with a low-PEEP strategy without recruitment maneuvers. In exploratory subgroup analyses, active smokers showed a numerically higher incidence of PPCs compared with non-smokers; however, this finding was based on a small number of events and should be considered hypothesis-generating. No clear interaction between smoking status and PEEP strategy was observed, suggesting that the effect of intraoperative PEEP within the tested range was not meaningfully modified by smoking status in this cohort.

These findings are consistent with previous randomized trials investigating intraoperative ventilation strategies. The PROBESE trial demonstrated that, in obese patients undergoing surgery under general anesthesia, higher PEEP combined with recruitment maneuvers did not reduce PPC incidence compared with lower-PEEP strategies [11]. Similarly, the PROVHILO trial showed no reduction in PPCs with higher PEEP levels in patients undergoing abdominal surgery, despite improvements in intraoperative oxygenation [12]. Our results are consistent with and further support these findings, suggesting that even in the presence of additional risk factors such as active smoking, increasing PEEP alone may not translate into clinically meaningful improvements in postoperative pulmonary outcomes.

The lack of benefit observed with the high-PEEP strategy may reflect the complex balance between alveolar recruitment and overdistension. From a pathophysiological perspective, obesity and active smoking may interact synergistically in promoting perioperative respiratory dysfunction. Obesity reduces functional residual capacity and chest wall compliance, while smoking contributes to airway inflammation, mucus hypersecretion, impaired mucociliary clearance, and small airway dysfunction [15]. Together, these mechanisms may increase susceptibility to atelectasis, ventilation–perfusion mismatch, and postoperative respiratory complications during general anesthesia. In smokers, the potential presence of airflow limitation and intrinsic PEEP may also influence the physiological response to externally applied PEEP, potentially contributing to heterogeneous effects of recruitment maneuvers and fixed high-PEEP strategies. While higher PEEP and recruitment maneuvers can reopen collapsed alveoli and improve oxygenation, they may also increase intrathoracic pressure, reduce venous return, and alter pulmonary perfusion, potentially offsetting beneficial effects. In obese patients, who are characterized by reduced functional residual capacity, increased chest wall elastance, and a high propensity for atelectasis formation, these competing physiological mechanisms are particularly relevant. Experimental and physiological studies have shown that non-individualized application of a high-PEEP strategy may lead to heterogeneous lung inflation, with simultaneous recruitment and overdistension in different lung regions [16,17].

The exploratory observation of a numerically higher PPC incidence among smokers is biologically plausible and supported by existing literature. Smoking is associated with airway inflammation, impaired mucociliary clearance, increased mucus production, and small airway dysfunction, all of which may predispose patients to postoperative respiratory complications [18]. These alterations may be particularly detrimental in obese patients, who already exhibit compromised respiratory mechanics. Previous studies have consistently identified smoking as a risk factor for PPCs, and perioperative smoking cessation has been associated with improved postoperative outcomes [19,20].

However, whether smoking modifies the response to intraoperative ventilatory strategies remains uncertain. Smoking-related airflow limitation and gas trapping could theoretically reduce the effectiveness of externally applied PEEP, particularly in the presence of intrinsic PEEP. In such cases, additional external PEEP may not significantly improve alveolar recruitment and could contribute to dynamic hyperinflation. Conversely, carefully titrated PEEP might be beneficial in selected patients. In the present study, intrinsic PEEP, driving pressure, and detailed respiratory mechanics were not assessed, limiting mechanistic interpretation. Therefore, any conclusions regarding the interaction between smoking status and ventilatory strategy should be considered speculative.

From a clinical perspective, our findings do not support the routine use of higher PEEP with recruitment maneuvers to prevent PPCs in obese surgical patients. The absence of a detectable benefit across both the overall population and exploratory subgroups suggests that a uniform high-PEEP strategy may not be appropriate. Recent literature increasingly supports individualized intraoperative ventilation approaches that integrate respiratory physiology and patient-specific characteristics rather than the uniform application of fixed PEEP levels across heterogeneous surgical populations [21]. Instead, these findings reinforce the importance of individualized ventilatory management and established perioperative strategies aimed at reducing pulmonary complications. Smoking cessation remains a key modifiable risk factor, and even short-term cessation may improve airway reactivity, while longer abstinence enhances mucociliary function and immune response [18,19].

These findings support the concept that fixed PEEP strategies may be insufficient in heterogeneous obese populations, particularly when smoking-related airway dysfunction is present, and reinforce the rationale for individualized, physiology-guided ventilatory approaches.

This study has several strengths, including its randomized design, prospective data collection, and standardized ventilatory protocols. The focus on obese patients enhances clinical relevance, as this population is at increased risk of PPCs. Furthermore, smoking status was prospectively recorded, allowing exploratory subgroup analyses.

**Limitations**. Nevertheless, several limitations must be acknowledged. First, the study was conducted at a single center with a relatively small sample size, limiting generalizability. Second, the number of PPC events was low, resulting in limited statistical power and wide uncertainty around effect estimates, particularly in subgroup analyses. A systematic institutional database specifically evaluating PPC incidence under comparable intraoperative ventilatory conditions was not available during the study period; therefore, retrospective institutional comparisons could not be reliably performed. Third, smoking status was based on self-report and categorized dichotomously, without detailed information on smoking exposure, such as pack-years or timing of cessation. The absence of smoking intensity and cessation data may have resulted in exposure misclassification, potentially biasing estimates toward the null. Smoking prevalence differed numerically between groups despite randomization, likely reflecting the modest sample size. Fourth, smoking was not a randomized variable, and residual confounding cannot be excluded. Fifth, perioperative management beyond ventilatory strategy was not fully standardized. Sixth, key physiological parameters such as intrinsic PEEP and driving pressure were not measured. Finally, although patient enrollment and data collection were completed between 2016 and 2018, additional time was required for data verification, exploratory analyses regarding smoking status, manuscript preparation, and revision.

Despite these limitations, the randomized prospective design, standardized ventilatory protocols, and consistency between unadjusted and exploratory adjusted analyses support the internal validity of the study findings. Furthermore, the present results are concordant with previous large randomized trials investigating intraoperative PEEP strategies in surgical patients.

## 5. Conclusions

In this randomized trial of obese surgical patients, a high-PEEP strategy with recruitment maneuvers did not reduce postoperative pulmonary complications compared with a low-PEEP strategy. Exploratory analyses suggested a numerically higher incidence of PPCs among active smokers; however, these findings should be interpreted cautiously given the limited number of events and the exploratory nature of the analyses.

Overall, the present findings are consistent with previous randomized studies and support the concept that routine application of fixed high-PEEP strategies may not improve postoperative pulmonary outcomes in obese surgical patients. Future larger studies incorporating detailed smoking exposure assessment and respiratory physiology monitoring are needed to better define individualized ventilation approaches.

## Figures and Tables

**Table 1:** Demographic characteristics of patients.

	Low PEEP	High PEEP	*p*-Value
**No. of patients**	45	50	
**M/F**	14/31	20/30	
**Age**	41.18 ± 10.26	39.54 ± 11.77	0.23926
**BMI**	45.74 ± 6.17	45.23 ± 5.66	0.340845
**Obesity class**			
**I**	0	0	
**II**	10	6	
**III**	25	34	
**IV**	10	8	
**V**	0	2	
**Smokers**	11 (24.4%)	17 (34%)	
**Ariscat score**	35.87 ± 4.78	38.5 ± 9.50	0.050033

Obesity classes were defined according to BMI categories established by the World Health Organization. Smoking status was classified according to self-reported active smoking at the time of preoperative evaluation. M, male; F, female; BMI, body mass index.

**Table 2:** Demographic characteristics of smoker patients.

	Low PEEP	High PEEP	*p*-Value
**No. of patients**	11	17	
**M/F**	3/8	9/8	
**Age**	36 ± 4.71	35.65 ± 5.69	0.435085
**BMI**	45.26 ± 5.27	44.69 ± 5.88	0.400856
**Obesity class**			
**I**	0	0	
**II**	2	3	
**III**	7	10	
**IV**	2	4	
**V**	0	0	
**Ariscat score**	36 ± 4.71	35.64 ± 5.69	0.435085

M, male; F, female; BMI, body mass index.

**Table 3:** Pulmonary complications.

Complication	*n*	%
Respiratory failure	3	50%
Atelectasis	1	17%
ARDS	1	17%
Pleural effusion	1	17%

## Data Availability

The data presented in this study are available on request from the corresponding author due to privacy restrictions and the presence of sensitive participant information. Data may be made available from the corresponding author upon reasonable request and subject to approval by the relevant ethics committee/institutional regulations.

## Supplementary Materials

The following supporting information can be downloaded at: https://www.mdpi.com/article/10.3390/medsci14020284/s1. Ref. [22] is cited in Supplementary Materials.

## Abbreviations

The following abbreviations are used in this manuscript:

ARDS	Acute Respiratory Distress Syndrome
ARISCAT	Assess Respiratory Risk in Surgical Patients in Catalonia
BMI	Body Mass Index
CI	Confidence Interval
COPD	Chronic Obstructive Pulmonary Disease
FiO_2_	Fraction of Inspired Oxygen
ICU	Intensive Care Unit
NYHA	New York Heart Association
PEEP	Positive End-Expiratory Pressure
PPCs	Postoperative Pulmonary Complications
RR	Relative Risk
SD	Standard Deviation
SpO_2_	Peripheral Oxygen Saturation
